# Correction: Microwave-assisted chemoselective synthesis and photophysical properties of 2-arylazo-biphenyl-4-carboxamides from hydrazonals

**DOI:** 10.1039/d3ra90102e

**Published:** 2023-10-26

**Authors:** Abdulrahman M. Alazemi, Kamal M. Dawood, Hamad M. Al-Matar, Wael M. Tohamy

**Affiliations:** a Chemistry Department, Faculty of Science, University of Kuwait P.O. Box 5969 Safat 13060 Kuwait a.alazmi@ku.edu.kw +965 24816482; b Department of Chemistry, Faculty of Science, Cairo University Giza 12613 Egypt kmdawood@sci.cu.edu.eg +202 35727556

## Abstract

Correction for ‘Microwave-assisted chemoselective synthesis and photophysical properties of 2-arylazo-biphenyl-4-carboxamides from hydrazonals’ by Abdulrahman M. Alazemi *et al.*, *RSC Adv.*, 2023, **13**, 25054–25068. https://doi.org/10.1039/d3ra04558g

The authors regret that an affiliation (affiliation *c*) was incorrectly included in the original manuscript. The corrected list of affiliations is as shown here.

The Royal Society of Chemistry apologises for these errors and any consequent inconvenience to authors and readers.

## Supplementary Material

